# Science to improve care for people affected by unhealthy alcohol and other drug use

**DOI:** 10.1186/1940-0640-7-1

**Published:** 2012-03-15

**Authors:** Richard Saitz, Jeffrey Samet

**Affiliations:** 1 Boston University Schools of Medicine and Public Health/Boston Medical Center, Boston, MA, USA

## Why *Addiction Science & Clinical Practice?*

The last 20 years have brought two major changes in the field of addiction. First, the US Institute of Medicine (IOM) encouraged recognition of a spectrum of alcohol and other drug use that affects health and is not limited to those with the highest severity [1]. Unhealthy use (the spectrum from use that risks consequences through addiction [2]) among those without addiction is much more common than addiction itself. The second major shift also began in the 1990s with an emphasis on addiction as a chronic illness [3,4] and on unhealthy use as a health condition.

Although drug and excessive alcohol use clearly have social and other environmental determinants and consequences, there is little doubt that health care should play a major role in addressing them. In the health-care sector, attention to unhealthy substance use cannot be limited to highly specialized care settings; most patients with these conditions appear in general health settings where such problems are all too often ignored. In 2006, the IOM urged improvements in the quality of care for substance use conditions [5]. Responding to that report is a major impetus for the establishment of *Addiction Science & Clinical Practice (ASCP)*. We see this focus as unique among journals that address addictions.

## History of *ASCP*

*Addiction Science & Clinical Practice *was founded in 2002 by the National Institute on Drug Abuse (NIDA), an agency of the US National Institutes of Health and the largest funder of drug-abuse research in the world. In 2011, NIDA discontinued ownership of the journal and transferred editorial control to us. We hope to see all of their subscribers and authors continuing the dialogue they established so well. Under NIDA's stewardship, *ASCP *excelled at bringing researchers and clinicians together to better translate science into practice and back again--to blend the two by publishing reviews and roundtables. We aim to take the next steps in this thematic direction: to serve as a forum for clinically relevant research and perspectives that contribute to improving the quality of care (prevention and treatment) for people with unhealthy alcohol, tobacco, or other drug use and addictive behaviors across a spectrum of clinical settings--from emergency departments to prisons, from primary-care offices to the Internet, and from general hospitals to specialty care programs.

## A modern publishing model for *ASCP*

To achieve these aims, we have moved firmly into the cutting edge of 21st-century publishing practices by teaming with BioMed Central. Although some feel tied to print publications, we realize that, in 2012, most scientific reading occurs online. We are interested in having a broad reach and large impact, and online open-access journals are best able to meet these goals. In this publishing model, authors pay the costs to publish their work, which funders of research recognize as legitimate and expected. For an initial period, we are grateful to have support from NIDA that allows us to defray those costs for authors.

With BioMed Central as publisher, *ASCP *continues to be listed in Medline, to have articles deposited in national and international repositories, and to make our content freely and permanently available around the globe at no charge. Articles will be published immediately upon acceptance and listed in PubMed shortly thereafter. Because they are widely indexed, articles in *ASCP *are easily and freely discoverable through Internet search engines. We seek to have an impact beyond the usual calculations of citations; we want our articles to benefit people receiving care.

## Building blocks

We appreciate the contributions of our exceptional group of Associate Editors and world-class Editorial Board who represent a range of disciplines (e.g., psychiatry, internal medicine, psychology), research approaches (e.g., clinical trials, health services, behavioral sciences), and countries and offer their expertise on a variety of substances (e.g., alcohol, drugs, tobacco).

We look forward to reviewing your quality work and appreciate in advance your willingness to peer review submissions that, in some dimension, will improve the quality of health care for individuals with unhealthy substance use. It is with great humility and honor that we accept the task of providing thoughtful, timely, and responsive reviews for all submissions to *ASCP *as it embarks on its next phase. We know all the articles we publish will benefit from the wisdom, creativity, and hard work of our international, multidisciplinary substance use research colleagues.
